# The Synergistic Refinement of Primary Si in Hypereutectic Al-Si Alloys Using Ce and Er Elements

**DOI:** 10.3390/ma19091901

**Published:** 2026-05-05

**Authors:** Zhendong Zhang, Shijie Li, Yakun Zhang, Guoqiang Lv, Zhengjie Chen, Kuixian Wei, Wenhui Ma

**Affiliations:** 1 Faculty of Metallurgical and Energy Engineering/National Engineering Research Center of Vacuum Metallurgy, Kunming University of Science and Technology, Kunming 650093, China; 18250183395@163.com (Z.Z.); li17366965298@163.com (S.L.); kxwei@kust.edu.cn (K.W.); mwhsilicon@126.com (W.M.); 2 State Key Laboratory of Complex Nonferrous Metal Resources Clean Utilization, Kunming University of Science and Technology, Kunming 650093, China; 3 School of Engineering, Yunnan University, Kunming 650500, China

**Keywords:** hypereutectic Al-Si alloys, grain refinement, primary Si, synergy, Ce and Er

## Abstract

Hypereutectic Al-Si alloys are used in a number of industries; however, the large size of primary Si grains significantly limits their industrial applications. Grain refinement through the addition of modifiers has become a crucial technical approach to overcome this challenge. Rare earth elements, particularly Ce and Er, are promising modifiers, and they have a synergistic effect on the refining of Si in hypereutectic Al-Si alloys. However, the synergistic mechanism between Ce and Er is rarely reported. Thus, this study aimed to reveal the mechanism underlying the synergistic effect of Ce and Er on refining the grains in hypereutectic Al-25 wt.% Si alloy. The results indicate that the synergistic doping of Ce and Er significantly reduces the size of the primary Si grains. The addition of 0.5 wt.% Ce and 1 wt.% Er to the alloy reduced the primary Si grain size to 428.51 μm, achieving an overall refinement rate of up to 55.1% compared with the alloy without Ce and Er. Microstructural analysis revealed that Ce and Er accumulated around the primary Si crystals, resulting in the formation of complex intermetallic phases. These intermetallic complex phases provided additional nucleation sites for the growth of primary Si, thereby promoting its heterogeneous nucleation and inhibiting grain growth. Furthermore, they reduced the intensity of crystal growth in the direction of the preferred growth orientation of the primary Si, thereby further inhibiting its growth. This study provides essential experimental evidence supporting the synergistic refinement of hypereutectic Al-Si alloys using Ce and Er.

## 1. Introduction

Rapid industrialization and economic growth have led to a steadily increasing demand for silicon materials. Among these materials, Al-Si alloys used in many fields due to their high specific strength, excellent castability, stable phase transition temperature, high solid–liquid latent heat density, good thermal stability, and superior electrochemical capacity and cycling performance when doped with other elements or systems [1,2,3]. Al-Si alloys play a crucial role in national economic development across sectors such as aerospace, electronics, electrical appliances, and building structural materials [4]. Based on their silicon content, Al-Si alloys can be classified into three types: hypoeutectic, eutectic and hypereutectic. Hypereutectic Al-Si alloys have a silicon content exceeding 12.6 wt.%. Owing to the high silicon content of hypereutectic Al-Si alloys, their mechanical properties are mainly governed by the primary silicon crystals [5,6]. Additionally, hypereutectic Al-Si alloys have a wide range of industrial applications owing to their excellent wear resistance, low thermal expansion coefficient, good heat resistance, high strength, outstanding castability, low density, and superior overall mechanical properties. In the automotive and aerospace industries, hypereutectic Al-Si alloys are widely used to manufacture components such as engine blocks, pistons and brake discs. Moreover, they have great application potential in several areas, such as thermal management and electronic packaging [7,8,9]. The two key factors in primary silicon—morphology and size—play a significant role in the properties of hypereutectic Al-Si alloys, thereby affecting their final performance. Primary Si significantly enhances the wear resistance and mechanical strength of the alloy. However, in hypereutectic Al-Si alloys produced using conventional casting methods (such as sand casting, die casting, and low-pressure casting), the primary Si grains are often excessively coarse, thereby reducing the overall mechanical properties of the Al-Si alloy. Therefore, controlling the size of primary Si grains is crucial for developing high-performance Al-Si alloy castings and expanding their applications [9,10,11]. Reducing the grain size enhances the properties of Al-Si alloys, making them more suitable for various applications [12]. Consequently, the effective refinement of primary Si in hypereutectic Al-Si alloys has become a key research focus in this field.

Currently, grains in alloys are commonly refined by (1) increasing the cooling rate of the alloy melt [13,14,15], (2) stirring the growing grains in the alloy [16,17,18], (3) applying vibration techniques [18,19,20], and (4) adding a certain amount of grain refiners or modifiers to the alloy [21,22,23]. However, the first three methods are not effective, making them unsuitable for industrial applications. In contrast, the addition of modifiers is a promising refining method owing to its controllable process and high efficiency. L. Bolzoni et al. [24] conducted refinement experiments on Al-Si alloys using niobium; their research revealed that the addition of these niobium-based intermetallic compounds significantly improved the microstructural characteristics of the Al-Si alloys under investigation. Zheng et al. [25] designed a multifunctional Al-5Ce-1B inoculant to refine the Al and eutectic Si phase. Their study found that the rich Ce clusters were present in two {111} Si. The adsorption at intersections and in the <112> Si growth direction could induce high-density multi-directional Si twins, enhance the isotropic growth of silicon and induce a phase transformation in eutectic silicon. Chen et al. [26] investigated the refinement effect of La-Ce-Y system in Al-25 wt.% Si alloy. The research results found that cerium has a more significant refining effect on primary Si than lanthanum, both can weaken the network distribution and induce the refinement of eutectic Si. Adding these two substances in combination can refine both primary silicon and eutectic silicon; in this case, the refining effect on primary silicon is far more pronounced than when they are added separately. Prabhkiran et al. [27] investigated the refinement effect of cerium oxide under electromagnetic stirring conditions on primary Si in Al-16 wt.% Si alloy. The results indicated that the incorporation of cerium oxide reduces the average particle size of the primary particles from 152 ± 9 μm to 120 ± 6 μm, achieving a maximum overall refinement rate of approximately 41.2%. Mario et al. [28] modified the Si phase by adding 1% Ce to the Al-8 wt.% Si alloy. The result showed that the Si phase was partially modified, and the eutectic solidification temperature was reduced by approximately 10 °C. Li et al. [29] refined the primary Si grains in Al-20 wt.% Si alloy by adding Er. The results showed that the addition of 0.5% Er significantly transformed the primary Si from coarse polygonal, plate-like, and star-like structures into fine block-like structures, while the eutectic Si transformed from coarse plate-like or needle-like structures into dense, coral-like fibrous structures. Additionally, the morphologies of both primary Si and eutectic Si were significantly improved under the modification effect of 0.5 wt.% Er. Xing et al. [30] prepared eutectic Al-12.6 wt.% Si alloys with different rare earth element Er contents using conventional casting techniques. The results of the study show that the addition of erbium (Er) significantly improves the alloy’s wear resistance and reduces its coefficient of friction. The addition of Er has altered the size and shape of the eutectic silicon, thereby refining the microstructure of the Al-Si alloy. Hu et al. [31] investigated the effect of rare earth erbium (Er) addition levels (0, 0.3, 0.6, and 0.9 wt.%) on the microstructure development and tensile properties of die cast ADC12 aluminum alloy. Research has found that the secondary dendrite arm spacing (SDAS) decreases with increasing Er content, and reaches its lowest point when the Er content increases to 0.6%. Furthermore, the structure of eutectic silicon changes from a coarse plate-like and needle-like structure to a fine branched and fibrous structure due to the incorporation of Er. Berkmen et al. [32] conducted studies on the refinement of Al-Si alloys, in which the AlP phase is regarded as an effective nucleation substrate. Faraj et al. [33] investigated the synergistic refinement effect of Ce and Er on the Al-7 wt.% Si alloy. The results showed that as Ce and Er contents increased, the size of the silicon intermetallic compounds in the alloy gradually decreased. This phenomenon indicated that the microstructure of the Al-7 wt.% Si alloy was significantly refined through the combined action of Ce and Er. Lei et al. [34] summarized research findings on the refinement effects of Sc and Zr, and found that the synergistic addition of Ce and Er was more effective than individual elements. Mario et al. [28] also found that the addition of 1% Ce to the Al-8 wt.% Si alloy partially modified the Si phase. The subsequent introduction of 0.04% Sr after Ce addition led to full modification of the Si phase in this alloy. Dong et al. [35] modified alloys with Sr, Yb, and the combined (Sr-Yb) system. The results of the study show that the morphology of eutectic silicon has changed from a plate-like structure to a finer, fibrous structure. Among these elements, the (Sr-Yb) synergistic addition system produced the most refined eutectic Si three-dimensional microstructure, displaying the optimal fibrous morphology. Cleiton Luiz Pereira et al. [12] also conducted studies on the refinement of bismuth (Bi) and antimony (Sb) in hypereutectic aluminum-silicon alloys. The research results show that although adding Sb can promote the growth of flake-like silicon and some fibrous forms of fragmented silicon, thereby increasing the solidification rate, Bi alloying maintains layered growth at all tested rates. In terms of achieving higher tensile performance, adding Bi is better than Sb, but the presence of Sb provides higher wear resistance. Prabhkiran Kaur et al. [36] also investigated the combined effects of electromagnetic stirring technology and the addition of rare earth elements lanthanum and the effect of doping with the rare earth elements lanthanum and manganese on the metallurgical and mechanical properties of high-silicon aluminum alloys. The results indicate that the coupled addition of lanthanum and manganese significantly improves the metallurgical properties of Al-Si alloys. In summary, both Ce and Er can effectively refine Al-Si alloys. Therefore, it is hypothesized that the simultaneous addition of Ce and Er to hypereutectic Al-Si alloys can produce a synergistic effect, thus enhancing refinement of primary Si. However, this hypothesis has not been confirmed or thoroughly explored.

Therefore, in this study, the refinement mechanism of primary Si in hypereutectic Al-25 wt.% Si alloy was investigated through the synergistic effects of Ce and Er. First, ImageJ (http://imagej.org) accessed on 3 October 2025 was used to measure the grain size of primary Si in alloys with varying Ce and Er concentrations, and the overall refinement rate of primary Si was subsequently calculated for each alloy. Then, the microstructures of alloys containing different Ce and Er contents were examined using scanning electron microscopy (SEM) to reveal how the grain structure of primary Si in Al-25 wt.% Si alloys changed with varying Ce and Er contents. The thermodynamic behavior that occurs during the heating process was explored using differential scanning calorimetry (DSC). Finally, wavelength dispersive spectrometry (WDS) and electron backscatter diffraction (EBSD) were used to examine the distribution of Ce and Er around the primary Si and the crystallographic data of the primary Si before and after modification, including crystal orientation and misorientation angles, thereby clarifying the synergistic refinement mechanism of Ce and Er on the primary Si in hypereutectic Al-Si alloys. This study provides important experimental data on the mechanism underlying the synergistic refinement of primary Si in hypereutectic Al-Si alloys.

## 2. Experimental Methods

### 2.1. Raw Materials

The raw materials used in this experiment were high-purity Al (99.99%), high-purity Si (99.999%), high-purity Ce (99.5%), and high-purity Er (99.99%). These raw materials were all supplied by Zhongnuo New Materials (Beijing) Technology Co., Ltd. These raw materials were all supplied by Zhongnuo New Materials (Beijing) Technology Co., Ltd. The hypereutectic Al-25 wt.% Si alloy containing Ce and Er was melted in an induction furnace. The crucibles used in the experiment were high-purity, dense Al_2_O_3_ crucibles (99% purity, inner diameter of 27 mm, outer diameter of 31 mm, height of 60 mm) and high-purity, dense graphite crucibles (99.9% purity, inner diameter of 32 mm, outer diameter of 40 mm, height of 70 mm).

### 2.2. Methods

**Figure 1:**
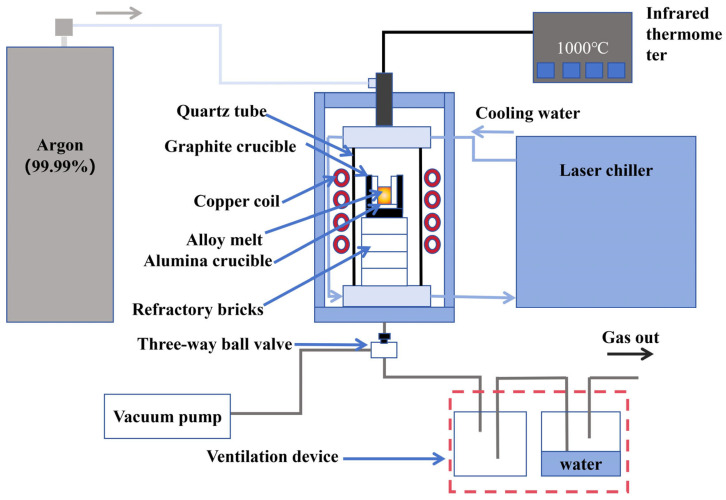
Medium frequency electromagnetic induction directional solidification furnace.

**Figure 2:**
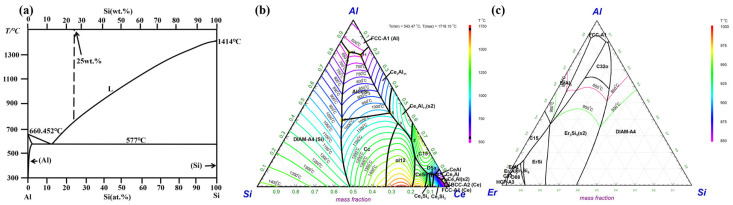
(**a**) The Al-Si binary system [37], (**b**) The Al-Si-Ce system, (**c**) The Al-Si-Er system.

Figure 1 shows the induction furnace used for alloy melting, comprising an induction coil, a vacuum system, a water tank and a positioning control system for directional solidification (the positioning control system is driven by a stepper motor). First, high-purity Al, Si, Ce, and Er were appropriately mixed to produce a compositionally uniform mixture. The mass ratio of Al to Si in the mixture was 3:1. The contents of Ce and Er (expressed as weight percentages of the total alloy mass) were set as follows: 0.0 wt.%, 0.5 wt.% Ce, 0.5 wt.% Ce-0.3 wt.% Er, 0.5 wt.% Ce-0.5 wt.% Er, 0.5 wt.% Ce-0.7 wt.% Er, 0.5 wt.% Ce-1 wt.% Er, 0.5 wt.% Ce-1.2 wt.% Er, 1 wt.% Ce, 1 wt.% Ce-0.3 wt.% Er, 1 wt.% Ce-0.5 wt.% Er, 1 wt.% Ce-0.7 wt.% Er, and 1 wt.% Ce-1 wt.% Er. Then, the mixed material was transferred into an Al_2_O_3_ crucible, then place the whole thing in a graphite crucible. The graphite crucible provided external support to prevent the Al_2_O_3_ crucible from cracking at high temperatures, thereby preventing contamination of the furnace chamber. Furthermore, Al_2_O_3_ crucibles cannot be heated by induction, while graphite crucibles exhibit excellent induction heating capabilities. As a result, graphite is used to assist heating in the induction furnace. Then, the crucible containing the assembled sample was placed in the heating zone of the induction furnace. Before heating, the air inside the quartz tube was evacuated using a vacuum pump at a pressure below 10^−2^ Pa. Then, argon gas with a purity of 99.99% was introduced to the system to prevent the oxidation of the hypereutectic Al-25 wt.% Si alloy during the melting process. This process was repeated at least three times, alternating between a vacuum and an argon-filled state, to ensure that any residual oxygen was completely removed. Subsequently, the sample was melted using induction heating. To ensure complete melting of the Al-Si-Ce-Er mixture, the melting temperature was set to 1273 K (well above the alloy’s melting point in Figure 2 [37], Figure 2b,c were generated using the Factsage software https://factsage.com; accessed on 22 April 2026) and held for 20 min. Once the holding phase is complete, the furnace current should be rapidly reduced to zero to allow the furnace to cool down under a flowing argon atmosphere, resulting in Al-25 wt.% Si alloys containing varying rare earth contents.

### 2.3. Analysis and Characterization

The solidified sample was cut into two pieces along a direction perpendicular to the bottom of the crucible. One piece was mechanically polished for analysis, and ImageJ was used to measure the size of the primary Si. The microstructure of the alloy was examined using SEM (JXA8230, JEOL, Tokyo, Japan). The thermodynamic behavior of the alloy was investigated using DSC (STA449F3, NETZSCH, Selb, Germany) at a heating rate of 10 °C/min. WDS (JXA8230, Japan) was used to analyze the distribution of rare earth elements Ce and Er. The crystal structure of primary Si was analyzed using EBSD (Sigma300, ZEISS, Oberkochen, Germany).

## 3. Results and Discussion

### 3.1. Refinement Effect Analysis

**Figure 3:**
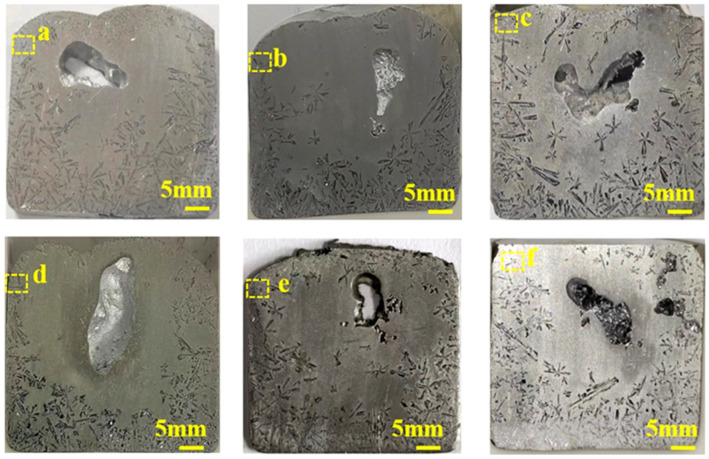
(**a**) Al-25 wt.% Si alloy, Al-25 wt.% Si alloy with different compositions of rare earths: (**b**) 1 wt.% Ce; (**c**) 1 wt.% Ce-0.3 wt.% Er; (**d**) 1 wt.% Ce-0.5 wt.% Er; (**e**) 1 wt.% Ce-0.7 wt.% Er; (**f**) 1 wt.% Ce-1 wt.% Er.

**Figure 4:**
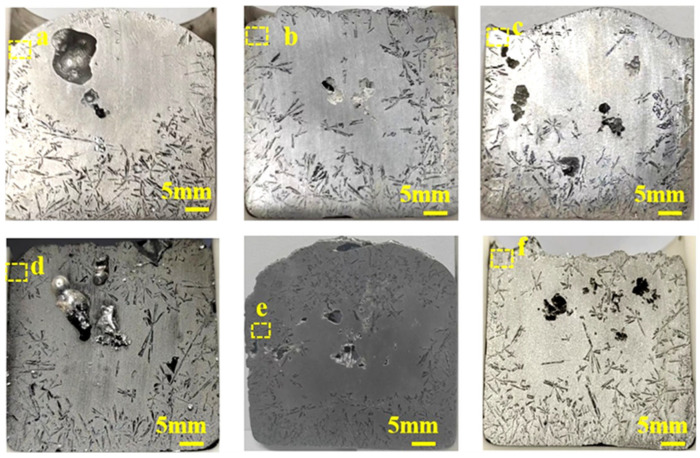
Al-25 wt.% Si alloy with different compositions of rare earths: (**a**) 0.5 wt.% Ce; (**b**) 0.5 wt.% Ce-0.3 wt.% Er; (**c**) 0.5 wt.% Ce-0.5 wt.% Er; (**d**) 0.5 wt.% Ce-0.7 wt.% Er; (**e**) 0.5 wt.% Ce-1 wt.% Er; (**f**) 0.5 wt.% Ce-1.2 wt.% Er.

**Figure 5:**
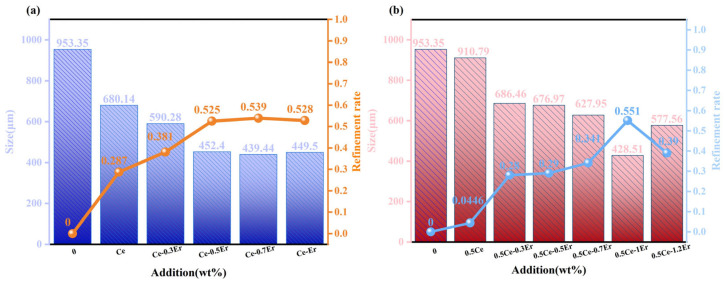
(**a**) Grain size variation and overall refinement rate of Al-25 wt.% Si alloy containing 1 wt.% Ce; (**b**) Grain size variation and overall refinement rate of Al-25 wt.% Si alloy modified with 0.5 wt.% Ce.

The average grain size of primary Si directly reflects the refinement effect of the alloy. Figure 3 and Figure 4 show images of hypereutectic Al-25 wt.% Si alloy samples containing varying Ce and Er contents. ImageJ software was used to analyze the grain size of alloys containing varying Ce and Er contents. Furthermore, the overall refinement rate was calculated to determine the refinement degree of primary Si in Al-25 wt.% Si alloys containing different rare earth contents. Figure 5 summarizes the primary Si grain size and the calculated overall refinement rate. The overall refinement rate $\eta_{r}$ can be expressed as:

(1) $$\eta_{r}={(l}_{0}-l_{i})/l_{0}$$

where $l_{i}$ is the average primary Si grain size (i.e., the length of primary Si) in the alloy with different rare earth additions, $l_{0}$ is the average primary Si grain size in the alloy without any rare earth addition. Figure 3a presents a photograph of the Al-25 wt.% Si alloy sample without Ce and Er. As shown in Figure 5a, the average size of primary Si in the Al-25 wt.% Si alloy without Ce and Er was 950 μm. Macroscopic examination revealed that the primary Si was extremely coarse. Figure 3b shows the actual sample of the Al-25 wt.% Si alloy modified with 1 wt.% Ce. The corresponding average size of primary Si and the overall refinement rate are presented in Figure 5a. As shown in Figure 5a, the average size of primary Si in the 1 wt.% Ce-modified alloy was reduced to 680 μm, achieving an overall refinement rate of 28.7%, indicating an initial refinement effect. As shown in Figure 3c–e, when the Ce content was fixed at 1 wt.%, the primary Si phase in the Al-25 wt.% Si alloy became progressively refined as the Er content increased from 0.3 wt.% to 0.7 wt.%. Moreover, the refinement ratio results in Figure 5a reveal that as the total rare earth content increases, the average grain size of primary Si has been steadily decreasing, thus gradually improving the refinement ratio. As shown in Figure 3e, the Al-25 wt.% Si alloy containing 1 wt.% Ce and 0.7 wt.% Er achieved the most effective refinement. The average size of the primary Si crystals was decreased to 439.44 μm, corresponding to an overall refinement rate of 53.9%. As shown in Figure 3f, when the content of Er exceeded 1 wt.% in the alloy modified with 1 wt.% Ce and 1 wt.% Er, the primary Si in the alloy did not undergo further refinement, instead, an increase in the average grain size was observed. As shown in Figure 5a, the average size of primary Si of the alloy modified with 1 wt.% Ce and 1 wt.% Er was 450 μm, achieving an overall refinement rate of 52.8%. Studies have attributed this effect to the poisoning interaction between the excessive contents of rare earth elements and the Si crystals [38,39,40]. The specific poisoning effect may be due to the mutual influence between the phases formed between Ce, Er, Al and the lattice of Si, which weakens the nucleation ability of Si phase. Just like the description of toxic effects in research of Li et al. [41]. Furthermore, according to TPRE theory, the atomic radius ratio of Ce to Si is 1.56, whilst that of Er to Si is 1.49. Like excellent modifiers such as Sr, these ratios are close to the theoretical atomic ratio of 1.64; theoretically, they should all exhibit excellent grain refinement effects, and their poisoning effects may also be attributable to the TPRE poisoning mechanism mentioned in some studies [42,43,44]. The pores within the structure are formed when the cooling rate is too rapid, causing the surface of the stirred melt to solidify before internal gases can escape. This phenomenon was also documented in the work of Gupta et al. [45].

The experimental results of the alloy modified with 0.5 wt.% Ce and incremental additions of Er are shown in Figure 4, and the corresponding refinement ratios and grain sizes are presented in Figure 5b. As shown in Figure 5b, the average size of primary Si in the 0.5 wt.% Ce-modified alloy was reduced to 911 μm, with a refinement ratio of only 4.46%. As the rare earth Er content increased from 0.3 wt.% to 1 wt.%, the average size of Si grains gradually decreased, and the overall refinement rate correspondingly improved. As shown in Figure 5b, the average size of primary Si of the alloy modified with 0.5 wt.% Ce and 1 wt.% Er was reduced to 428.51 μm, achieving an overall refinement rate of 55.1%. The best refinement was achieved under a fixed Ce content of 0.5 wt.%. However, when the content of Er was 1.2 wt.%, the grain size increased to 577.56 μm rather than decreased, as a result, the overall refinement rate decreased to 39%. This phenomenon indicates that an excessive amount of Er at this stage also has a poisoning effect on the Si crystals. By comparing all the above results, the alloy containing 0.5 wt.% Ce and 1 wt.% Er achieved the optimal grain refinement effect.

**Figure 6:**
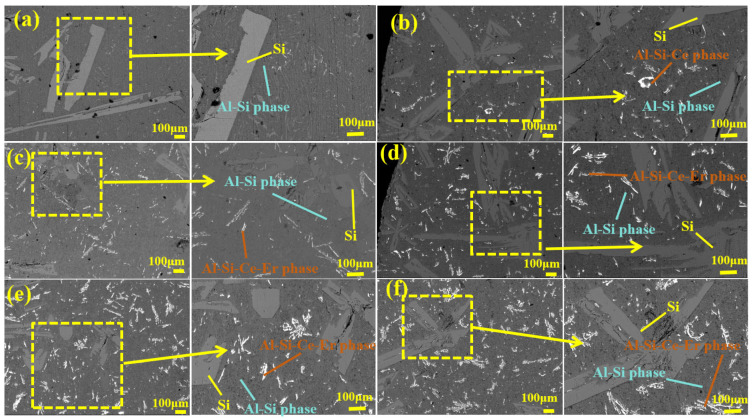
(**a**) SEM of Al-25 wt.% Si alloy; (**b**) SEM of Al-25 wt.% Si-1 wt.% Ce alloy; (**c**) SEM of Al-25 wt.% Si-1 wt.% Ce-0.3 wt.% Er alloy; (**d**) SEM of Al-25 wt.% Si-1 wt.% Ce-0.5 wt.% Er alloy; (**e**) SEM of Al-25 wt.% Si-1 wt.% Ce-0.7 wt.% Er alloy; (**f**) SEM of Al-25 wt.% Si-1 wt.% Ce-1 wt.% Er alloy.

SEM images of the Al-25 wt.% Si alloy containing 1 wt.% Ce and the Al-25 wt.% Si alloy without Ce and Er are shown in Figure 6. As illustrated in Figure 6a, the primary Si in the hypereutectic Al-Si alloy without any rare earth predominantly appeared as coarse columnar and elongated structures. Figure 6b–f show SEM images of alloys modified with 1 wt.% Ce. After the alloy was synergistically modified with Ce and Er, most of the relatively thick and elongated primary Si were gradually transformed into fine spherical, dendritic, and irregular shapes. As shown in Figure 6b–f, the primary Si phase in the 1 wt.% Ce-modified Al-25 wt.% Si alloy was progressively refined as the Er content increased from 0.3 wt.% to 0.7 wt.%. The observed trend in microstructural evolution indicates that increasing rare earth concentration progressively decreases the size of the primary Si phase and produces finer morphology. The results showed that the Al-25 wt.% Si alloy modified with 1 wt.% Ce and 0.7 wt.% Er (Figure 6e) exhibited the optimal refinement, and most of the coarse primary Si was transformed into fine primary Si grains. As shown in Figure 6f, further refinement of primary Si did not occur when the content of Ce and Er was 1 wt.% each. However, some of the fine grains were transformed into coarse columnar structures. As shown in Figure 6f, the size of the primary Si in the alloy modified with 1 wt.% Ce and 1 wt.% Er increased, and its particles became coarser than those of the alloy modified with 1 wt.% Ce and 0.7 wt.% Er. This observation is consistent with the poisoning effect mechanism [38,39,40].

**Figure 7:**
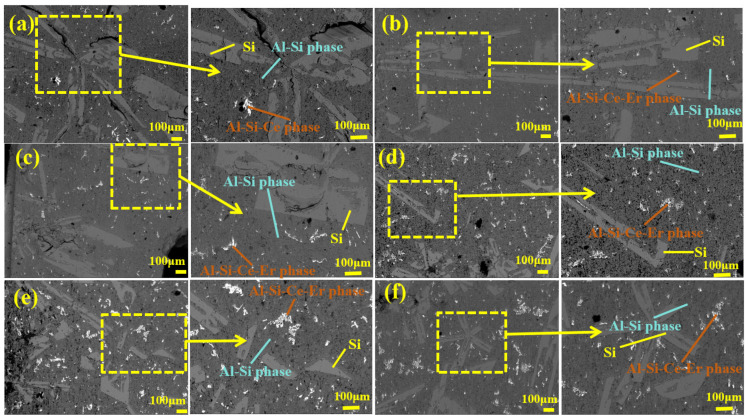
(**a**) SEM of Al-25 wt.% Si-0.5 wt.% Ce alloy; (**b**) SEM of Al-25 wt.% Si-0.5 wt.% Ce-0.3 wt.% Er alloy; (**c**) SEM of Al-25 wt.% Si-0.5 wt.% Ce-0.5 wt.% Er alloy; (**d**) SEM of Al-25 wt.% Si-0.5 wt.% Ce-0.7 wt.% Er alloy; (**e**) SEM of Al-25 wt.% Si-0.5 wt.% Ce-1 wt.% Er alloy; (**f**) SEM of Al-25 wt.% Si-0.5 wt.% Ce-1.2 wt.% Er alloy.

Figure 7 displays the SEM images of the Al-25 wt.% Si alloy with 0.5 wt.% Ce addition. As observed in Figure 7a, the primary Si phase in the Al-25 wt.% Si alloy with 0.5 wt.% Ce addition still exhibits several coarse columnar and elongated morphologies. Figure 7b–f show SEM images of alloys with modified 0.5 wt.% Ce. After the synergistic modification of the alloy with Ce and Er, most of the coarse columnar and elongated primary Si crystals were gradually transformed into fine spherical, dendritic, and irregular shapes. As shown in Figure 7b–e, with the Er content increased from 0.3 wt.% to 1 wt.%, the primary Si phase in the Al-25 wt.% Si alloy modified with a constant 0.5 wt.% Ce is gradually refined. The observed trend in microstructural evolution indicates that as the rare earth element content increases, the size of the primary Si continues to decrease, resulting in a more uniform morphology. The results showed that the Al-25 wt.% Si alloy modified with 0.5 wt.% Ce and 1 wt.% Er (Figure 7e) achieved the optimal refinement. In its microstructure, the majority of primary Si grains evolved from coarse columnar and elongated morphologies into refined spherical shapes, dendritic, and irregular shapes. As shown in Figure 7f, the primary Si in the alloy modified with 0.5 wt.% Ce and 1.2 wt.% Er did not undergo further refinement as Er content further increased; instead, the particles became coarser. As shown in Figure 7f, the primary Si size in the alloy containing 0.5 wt.% Ce and 1.2 wt.% Er was significantly larger than that in the alloy containing 0.5 wt.% Ce and 1 wt.% Er. This observation aligns with the established mechanism of the poisoning effect [38,39,40]. The above SEM images at different magnifications show that in both alloy groups containing 0.5 wt.% and 1 wt.% Ce, the combined addition of Ce and Er results in a pronounced grain refinement effect. Although both groups exhibited a poisoning phenomenon when Ce and Er contents were in excess, the synergistic grain refinement effect of Ce and Er remained pronounced.

### 3.2. Mechanism Refinement Analysis

To further elucidate the synergistic refinement effect and underlying mechanism of Ce and Er addition on primary silicon in hypereutectic Al-25 wt.% Si alloys, detailed analyses were performed on the element distribution, phase composition, and crystal structure of the hypereutectic Al-25 wt.% Si alloy.

**Figure 8:**
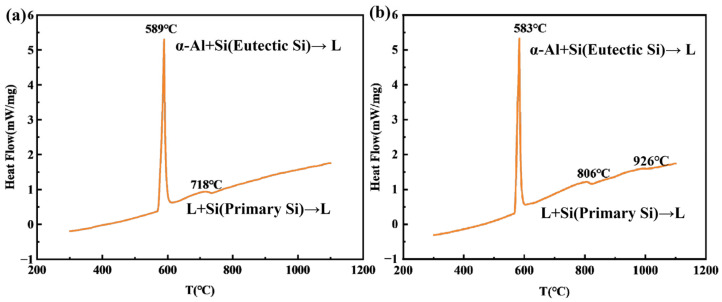
(**a**) DSC image of Al-25 wt.% Si alloy; (**b**) DSC image of Al-25 wt.% Si-0.5 wt.% Ce-1 wt.% Er alloy.

Figure 8 shows the DSC results obtained for the Al-25 wt.% Si alloy with and without Ce and Er. Figure 8a presents the DSC curve of the Al-25 wt.% Si alloy during heating, and Figure 8b presents the DSC curve of the Al-25 wt.% Si-0.5 wt.% Ce-1 wt.% Er alloy during heating. The results showed that two consecutive endothermic peaks were observed in both the Al-25 wt.% Si alloy and the Al-25 wt.% Si-0.5 wt.% Ce-1 wt.% Er alloy within the temperature range of 200–1200 °C. The peak temperatures for the Al-25 wt.% Si alloy were 589 °C and 718 °C, while those for the Al-25 wt.% Si-0.5 wt.% Ce-1 wt.% Er alloy were 583 °C and 806 °C. For both alloys, the two peaks at 589 °C and 583 °C correspond to the appearance of the binary Al-Si eutectic phase. The peak at 718 °C in the Al-25 wt.% Si alloy indicates the precipitation of primary Si. However, the peak temperature of the Al-25 wt.% Si-0.5 wt.% Ce-1 wt.% Er alloy observed at 806 °C was attributed to the addition of Ce and Er, which thereby changed the nucleation temperature of primary Si. The small peak near 1000 °C in the DSC detection results of Figure 8b is the rare earth phase, this situation is also similar to the situation in the work of Mario De Giovanni et al. [28]. Their research revealed that an intermetallic compound (i.e., a rare earth phase) appeared after the first peak. Since the actual nucleation temperatures of primary silicon in these two systems are similar and relatively low, the presence of rare-earth elements reduces the undercooling of primary silicon. This reduced undercooling promotes rapid nucleation, thereby leading to grain refinement. This is consistent with the results reported by Wang et al. [46].

**Figure 9:**
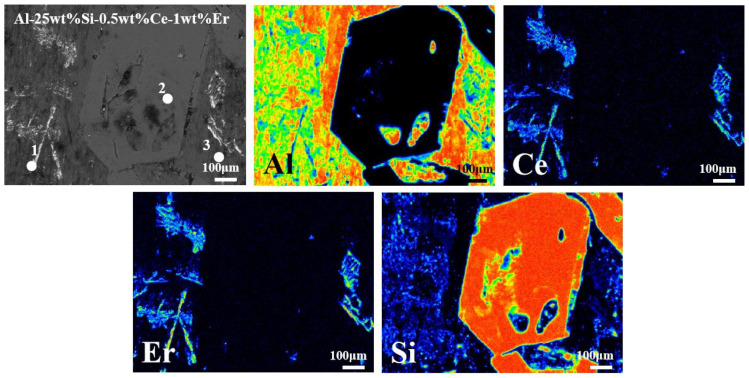
EPMA-mapping diagram of Al-25 wt.% Si-0.5 wt.% Ce-1 wt.% Er alloy.

The role of heterogeneous nucleation sites on primary Si was examined using the EPMA-mapping results of the distribution of primary Si and rare earth phases. This examination was conducted to confirm the formation of heterogeneous nucleation sites of Ce and Er near the solid–liquid interface of primary Si. The electron probe micro-analyzer (EPMA) mapping results (Figure 9) revealed that the Al-25 wt.% Si-0.5 wt.% Ce-1 wt.% Er alloy exhibited the best refinement effect in this study. The point analysis results (Table 1) showed that the white phase surrounding the primary Si crystals corresponded to a rare earth-enriched phase containing Ce and Er. Through WDS mapping analysis, it was found that Ce and Er showed obvious overlap and enrichment in the vicinity of primary Si crystals, thereby promoting the formation of Ce and Er-enriched phases. This result is consistent with the point analysis. These findings show that the Ce and Er-enriched phases serve as heterogeneous nucleation sites for primary Si crystals, thereby suppressing their growth at the solid–liquid interface. Li et al. [47] reported that the addition of modifiers led to the creation of heterogeneous nucleation sites, resulting in a significant grain refinement effect. Therefore, the formation of Ce and Er-enriched phases facilitated grain refinement. Similar complex intermetallic phases enriched with rare earth elements were reported by Saleh et al. [48]. Zhang et al. [49] further revealed that Er-enriched phases were distributed near the solid–liquid interface of Si, exhibiting an inhibitory effect on Si.

The results of DSC and EPMA revealed that Ce and Er form rare-earth-enriched phases near the primary Si crystals. These enriched phases served as heterogeneous nucleation sites, significantly decreasing the undercooling and thereby promoting the rapid nucleation of primary Si crystals. Meanwhile, the enriched phases significantly inhibited the subsequent growth of these crystals. Moreover, the synergistic effect of Ce and Er in altering the growth behavior of primary Si was a key factor in suppressing its growth.

**Figure 10:**
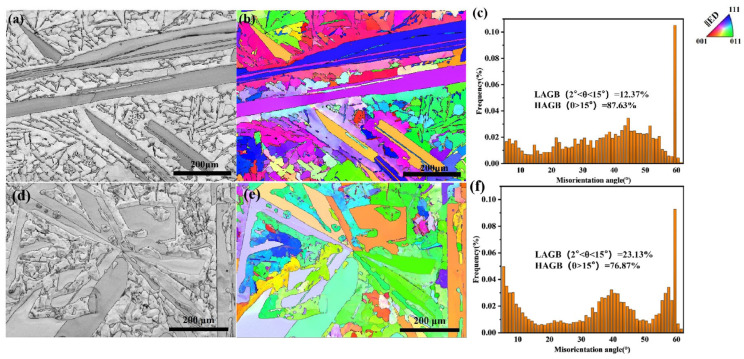
EBSD diagram of Al-Si alloy: (**a**) EBSD microstructure map of the Al-25 wt.% Si alloy; (**b**) EBSD orientation map of the Al-25 wt.% Si alloy; (**c**) EBSD misorientation map of the Al-25 wt.% Si alloy; (**d**) EBSD microstructure map of the Al-25 wt.% Si-0.5 wt.% Ce-1 wt.% Er alloy; (**e**) EBSD orientation map of the Al-25 wt.% Si-0.5 wt.% Ce-1 wt.% Er alloy; (**f**) EBSD misorientation map of the Al-25 wt.% Si-0.5 wt.% Ce-1 wt.% Er alloy.

Thus, EBSD was used to analyze the growth behavior of primary Si, and the analysis was performed using ATEX [50]. The observation plane for EBSD and micrograph analysis is a longitudinal cross-section of the specimen parallel to the solidification direction. As shown in Figure 10, the EBSD morphology of the unmodified Al-25 wt.% Si alloy is depicted specifically in Figure 10a. In Figure 10a, the grey scale represents the quality of the diffraction pattern; bright areas indicate high crystal integrity, whilst dark areas correspond to grain boundaries or defects. This is a BC diagram; like the IQ diagram, it reflects the quality of the crystal diffraction. The crystal orientation diagram contains only silicon; through phase selection, it excludes aluminum and rare earth elements. The result showed that the primary Si crystals were relatively large, elongated, and exhibited a single growth orientation. The crystallographic orientation results of the unmodified Al-25 wt.% Si alloy without any rare earth additions are presented in Figure 10b. The results indicated that the primary Si crystals in the Al-25 wt.% Si alloy mainly exhibited a singular crystallographic orientation, indicating anisotropic growth. Comparative orientation analysis revealed that this orientation was mainly aligned along the <111> direction. The <111> direction aligns with the normal to the observation plane, indicating that unmodified primary crystalline Si exhibits strong <111> preferential orientation. As <111> is the primary crystalline Si’s predominant preferential growth direction, this results in anisotropy and a tendency towards grain coarsening and growth. To determine the differences in the primary Si orientation before and after modification, an analysis of the orientation difference angles was performed. The results for the orientation difference angles of the Al-25 wt.% Si alloy before modification are presented in Figure 10c. The results revealed that the unmodified Al-25 wt.% Si alloy exhibited a higher frequency of orientation difference angles near 60°.

**Figure 11:**
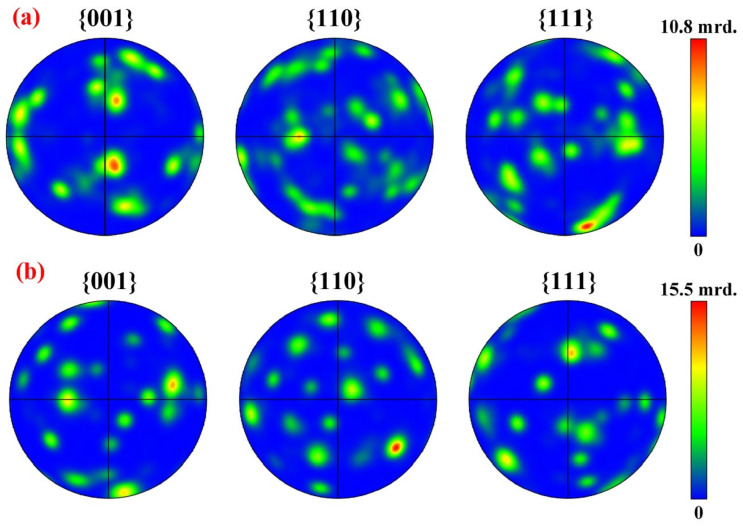
(**a**) Pole figures of the Al-25 wt.% Si alloy; (**b**) Pole figures of the Al-25 wt.% Si-0.5 wt.% Ce-1 wt.% Er alloy.

Figure 10d shows the EBSD microstructure of the modified Al-25 wt.% Si-0.5 wt.% Ce-1 wt.% Er alloy. Compared with the primary Si in the unmodified Al-25 wt.% Si alloy (Figure 10a), the morphology and size of the primary Si in the modified alloy were significantly altered. Additionally, the growth direction of the primary Si was changed, displaying a more irregular and disordered orientation. The crystallographic orientation results of primary Si in the rare earth-modified Al-25 wt.% Si-0.5 wt.% Ce-1 wt.% Er alloy are presented in Figure 10e. Following the co-addition of Ce and Er, preferential orientation has been observed in the crystal structure along the <110> and <111> directions. Compared with Figure 11a, it is clear that the orientation dispersion of the initial Si increased, the concentration of the polar graph decreased, and the preferential orientation was significantly weakened. The orientation difference angle results for the modified Al-25 wt.% Si-0.5 wt.% Ce-1 wt.% Er alloy are presented in Figure 10f. The results indicated a reduction in high-angle grain boundaries (HAGB), an increase in low-angle grain boundaries (LAGB), as well as a leftward shift of the HAGB distribution. This phenomenon indicates a decrease in the orientation difference between adjacent primary Si crystals after modification. In the unmodified Al-25 wt.% Si alloy, the primary Si exhibited growth behavior that is almost anisotropic. The primary Si in the modified Al-25 wt.% Si-0.5 wt.% Ce-1 wt.% Er alloy grew in a more isotropic manner. This variation can be ascribed to the synergistic refining effect of Ce and Er, which restrains the growth of primary Si in the Al-25 wt.% Si alloy and progressively decreases its grain size. This result is consistent with the results reported by Manuel et al. [51] and Tan et al. [52], where the modified primary Si exhibited isotropic growth, whereas the unmodified primary Si showed anisotropic growth. These results are also supported by the findings of G. Marinelli et al. [53]. Manuel et al. reported that grain refinement has the potential to generate randomly oriented crystallographic grains, thereby facilitating isotropic material behavior.

Figure 11 depicts the pole figures of two alloy groups. As presented in Figure 11a, the pole figure of the unmodified alloy shows a prominent texture. It shows the crystal orientation is not random but exhibits a distinct preferential orientation. In the {100} and {110} pole figures, regions with a high concentration of poles of relatively high intensity can be observed. This phenomenon indicates that during the natural growth of primary Si, it tends to grow rapidly along specific crystallographic directions, thereby exhibiting anisotropic growth characteristics. In contrast, Figure 11b depicts the 0.5 wt.% Ce-1 wt.% Er modified alloy. The pole figure exhibits relatively weak overall intensity for the growth orientation and a highly scattered distribution of poles. There are no concentrated high-intensity regions similar to those in Figure 11a. This implies that the crystal orientation of primary Si has become randomized, and there is no longer an obvious preferential orientation. Clearly, the addition of Ce and Er suppresses the anisotropic growth of primary Si, causing its growth behavior to approach isotropy. This result provides strong crystallographic evidence in support of the findings from a series of tests, including EPMA and growth orientation mapping in EBSD. The enrichment phases formed by Ce and Er elements around the primary Si crystals effectively impede the attachment of silicon atoms to the crystal planes with the lowest energy and the fastest growth rate (typically the {111} plane). Once the fastest growth direction is inhibited, the growth rates of the crystals in all directions become relatively balanced. As a result, the growth morphology changes from coarse plate-shaped and strip-shaped forms to fine blocky and spherical morphologies. As shown in Figure 11b, the growth is no longer confined to a few specific orientations, and the crystal orientation becomes randomized. Since growth is inhibited and occurs simultaneously in all directions, the crystals do not have sufficient time and space to develop into coarse particles. Eventually, grain refinement is achieved.

According to the nucleation process, heterogeneous nucleation sites can decrease the degree of undercooling and reduce the required nucleation energy. In the absence of rare earth elements in the alloy, the nucleation of silicon crystals occurred only as a result of energy fluctuations. Nevertheless, the introduction of Ce and Er led to the generation of heterogeneous nucleation sites, thereby suppressing the growth of primary silicon crystals. During this process, compounds rich in Ce and Er will preferentially precipitate prior to primary silicon due to their high melting points. The small peak observed around 1000 °C in the differential scanning calorimetry (DSC) results correspond to the exothermic peak of these cerium and erbium-enriched compounds. Therefore, during the cooling process, the rare earth phase first solidifies and forms heterogeneous nucleation sites around the Si crystal, and continues to participate in this process, inhibiting the growth of primary silicon crystals. This effect is reflected in the DSC results, where the synergistic addition of Ce and Er decreases the undercooling of the system. When silicon grains nucleate at lower undercooling, their grain size further decreases. In addition, due to the formation of wetting angles between solutes and the increase in nucleation sites, the critical nucleation work during heterogeneous nucleation is further reduced. In contrast, the critical nucleation work for homogeneous nucleation was greater, which enabled Ce and Er, acting as heterogeneous nucleation sites, to be adsorbed more extensively around the primary Si crystals. Through this process, the bonding between Si grains was reduced, which effectively restricted the growth of Si crystals.

This series of processes ultimately led to a reduction in the size of the solidified Si grains. This finding is consistent with the analytical results from various tests conducted on Al-25 wt.% Si alloys containing different amounts of rare earth additions mentioned above. This consistency indicates that Ce and Er have a refining and modifying effect on the primary Si in Al-25 wt.% Si alloys.

## 4. Conclusions

In this study, the introduction of Ce and Er to the Al-25 wt.% Si alloy led to the refinement of primary Si through their synergistic effect. Based on the experimental results, the following conclusions were drawn:

The alloy doped with 0.5 wt.% Ce and 1 wt.% Er exhibited the most significant refining effect on primary Si crystals, as the size of primary Si in the alloy was reduced from 953 μm to 429 μm, realizing a remarkable reduction in grain size, achieving an overall refinement rate of 55.1%. Microstructural analysis demonstrated that the synergistic effect of Ce and Er refined the morphology of primary Si in the alloy.

The Ce and Er elements generated solute-rich phases in the vicinity of primary Si crystals, offering heterogeneous nucleation substrates adjacent to the solid–liquid interface. This phenomenon decreased the degree of undercooling, suppressed the growth of the primary Si crystals.

In contrast to the alloy without Ce and Er doping, the modified alloy with these two elements exhibited a significant change in the growth orientation of primary silicon crystals—greatly suppressing their preferred growth orientation. This change effectively inhibited the growth of primary silicon, thereby realizing the refinement of primary silicon crystals.

## Figures and Tables

**Table 1:** Chemical composition content of each point in the EPMA, wt.%.

	Al	Si	Ce	Er
Point.1	36.5	21.15	13.26	29.1
Point.2	—	100	—	—
Point.3	77.54	22.46	—	—

“—” indicates below the EPMA detection limit.

## Data Availability

The original contributions presented in this study are included in the article. Further inquiries can be directed to the corresponding authors.
